# The Scottish Hepatology Access Research Partnership (SHARP) improving access to liver services throughout Scotland

**DOI:** 10.3310/nihropenres.13650.1

**Published:** 2024-10-10

**Authors:** Ruairi Lynch, Jonathan Fallowfield, David Blane, Rachael Swann, Kirsty Mills, Amy Cordwell, Ewan Forrest, Scottish Hepatology Access Research Partnership (SHARP)

**Affiliations:** 1 Department of Hepatology, NHS Tayside, Dundee, Scotland, UK; 2 Institute for Regeneration and Repair, The University of Edinburgh, Edinburgh, Scotland, UK; 3 University of Glasgow, Glasgow, Scotland, UK; 4 NHS Greater Glasgow and Clyde, Glasgow, Scotland, UK; 5 Patient representative, Inverness, UK; 6 British Liver Trust, Ringwood, England, UK

**Keywords:** Hepatology, access, socioeconomic deprivation, partnership, chronic liver disease

## Abstract

**Background and aims:**

Scotland has the highest rate of deaths from chronic liver disease (CLD) in the UK. Socioeconomic and geographic isolation represent significant challenges to delivery of care. The multidisciplinary Scottish Hepatology Access Research Partnership (SHARP) aimed to identify and break down barriers to diagnosing and treating liver disease in Scotland.

**Methods:**

SHARP comprised a core Partnership Management Group that developed projects and a Partnership Advisory Group which provided oversight.

**Results:**

SHARP established workstreams to achieve its aims:

Understanding current access to liver services

To identify barriers to liver patient care in Scotland we audited liver services and surveyed the experience of patients (n=276); primary care physicians (n=199) and Gastroenterologists/Hepatologists (n=99).

Technologies to monitor and diagnose CLD

Liver disease is diagnosed and monitored using routine blood testing which disadvantages isolated patients. We plan to develop a point of use test to analyse ALT and AST to enable community-based identification and monitoring of liver disease.

Identification of patients at risk of liver disease

CLD is often diagnosed late. We propose developing an artificial intelligence tool to predict an individual's risk of an emergent admission to hospital due to CLD. This tool will be validated in a Welsh cohort.

Barriers to engagement with care for liver disease

Hepatology did-not-attend rates are the highest of any specialty. We propose research to co-design a suite of recommendations to improve engagement with care for CLD patients. We aim to achieve this by interviewing practitioners alongside patients who do and don’t engage with services.

**Conclusions:**

Through a national survey SHARP has developed an understanding of the issues affecting access to hepatology services in Scotland. SHARP has developed projects that will help address the issues that socioeconomically and geographically isolated patients face when it comes to identifying and treating liver disease.

## Plain Language Summary

People may only realise they have liver disease when they get severe illness as a result of liver failure which may lead to death. The liver can be damaged over a long time before the person with liver disease is aware of anything being wrong. Many people with liver disease have difficulty getting to see doctors and specialist medical services. This may be due to a bad personal or social situation such as living in poorer deprived areas, or perhaps because they live in places where there are fewer medical services. The Scottish Hepatology Access Research Partnership (SHARP) project wanted to understand and improve access to liver services in Scotland.

SHARP included people from all over Scotland. We included specialists in liver disease and public health, general practitioners, social care services, charities and patient representatives through the British Liver Trust.

At the start of SHARP, we did a survey of patients and doctors to find what issues affect the care provided to patients with liver disease. With this information we have designed projects to help address the issues found. We know that patients can find it hard to access doctors and one project is trying to develop blood tests that can be done in people’s homes. This test will use a fingerprick of blood to identify liver damage. Another project aims to find patients before they get severe illness by using artificial intelligence. This project will use health records to find patients that are at risk of having liver disease. The final project we have developed will look at what stops patients attending clinic appointments. This project will develop tools to help improve the patient experience.

## Introduction

Deaths from chronic liver disease (CLD) in the UK are among the highest in Western Europe and Scotland has the highest rate among the four nations ( ScotPHO, 2015). However, within Scotland, there are significant regional variations, with areas of high social deprivation (Greater Glasgow & Clyde, Lanarkshire, and Forth Valley) and those with remote and rural populations (Highland including the Western Isles), which have particularly high rates of CLD deaths ( ScotPHO, 2020).

More than 80% of CLD deaths in Scotland are due to alcohol-related liver disease (ARLD) and these deaths occur disproportionately in the most socioeconomically disadvantaged sections of society. The challenges of accessing care for people experiencing severe disadvantages are multiple, especially for those who are homeless or struggle to engage with traditional models of care. Furthermore, the detection and management of an asymptomatic condition may not rate highly in these individuals’ personal or health priorities, yet early identification and intervention are imperative to prevent future morbidity and mortality ( Karlsen *et al.*, 2022; Williams *et al.*, 2020).

Socioeconomic deprivation is perhaps most obvious in an urban environment; however, it can also exist in remote and rural areas, where geographical isolation provides challenges in the identification and delivery of care for all etiologies of liver disease. This is supported by a report from the Scottish Health Action on Alcohol Policy (SHAAP), which examined alcohol use in rural Scotland ( MacDiarmid, 2020). It was found that expensive, lengthy, and infrequent public transport links and limited internet service were significant challenges for healthcare providers. Furthermore, alcohol-related harm was found to be disproportionate in rural communities, as alcohol is entrenched in social norms due to traditions, hospitality, and economic dependence on tourism and alcohol production. The report recommended that research with general practitioners support patients in rural communities, as primary care is often the first contact for those seeking help.

To address the geographical and socioeconomic challenges of identifying and managing people with liver disease in Scotland, we established the Scottish Hepatology Access Research Partnership (SHARP). SHARP is a multidisciplinary partnership with representation from throughout Scotland that, in partnership with patient representatives, has developed projects that aim to transform the care of patients with liver disease and improve health outcomes. SHARP aimed to develop projects that would help identify and break down the barriers that both geographically and socioeconomically isolated patients face when identifying and treating liver disease.

## Methods

### Patient and Public Involvement (PPI)

The British Liver Trust were a co-applicant on the SHARP grant and have been key members of the development of projects. In addition, a patient partner and representation from the Simon Community and Waverley Care were involved in an advisory role in the development of projects. Patient and carer feedback was sought on the proposed projects being developed by SHARP, the results of which are included in the results section. Patient and carer opinions were also sought as part of the survey of access to liver services.

### Structure

SHARP was led by Dr Ruairi Lynch from NHS Tayside and Prof. Ewan Forrest from NHS Greater Glasgow and Clyde (GG&C). To ensure effective representation and oversight, SHARP comprised a core Partnership Management Group (PMG) and an overseeing Partnership Advisory Group (PAG). The time for members of the PMG was funded by the NIHR Grant (award ID: NIHR155593) and included Hepatologists, representatives from Primary Care (including the Deep End practices that represent those from the most deprived areas in Scotland), Liver Nurse Specialists, Public Health, and Social Work (Alcohol and Drug Partnerships [ADP]). In addition, patient representation was included in the PMG, with representation from the British Liver Trust (BLT). The PMG met monthly, working groups were formed within the PMG, and these groups met more frequently to develop specific workstreams. The outputs from these working groups are outlined below in the results section. All members of the PMG were co-applicants on the grant and there was representation from throughout Scotland: NHS Tayside, NHS GG&C, NHS Lothian, NHS Lanarkshire, NHS Dumfries and Galloway, NHS Highland, NHS Western Isles and NHS Grampian. 

The variety of specialists involved in the PMG was mirrored in the PAG with an equally broad geographical representation. A list of members of each group is provided in the Appendix. Additionally, patient involvement in the PMG was bolstered by a co-opted patient partner and representation from the Simon Community and Waverley Care. The PAG was involved in meetings every three months and provided feedback on the evolving projects.

### Administration and management

The project was administered by the NHS GG&C Clinical Trials Unit (CTU). Trial design and statistical support were provided by the Robertson Centre for Biostatistics, with Professor John Petrie serving as a co-applicant.

### Meeting logistics

To ensure the involvement of representatives from Scotland, meetings were held virtually using Microsoft Teams. Hosting meetings virtually also ensured that the carbon footprint of SHARP was minimised.

### Surveys

Multiple surveys were conducted throughout the partnership period. All surveys were promoted through the SHARP social media channels and with press releases from NHS Research Scotland. Additionally, surveys were promoted to patients by the BLT using social media platforms and established patient networks. Finally, surveys targeting physicians were promoted using clinical networks such as the Scottish Society of Gastroenterology and the Deep End network of general practices. Surveys were administered via two separate platforms.

- A survey on the relevance of the SHARP workstreams to patients, the public, and clinicians was conducted using Microsoft Forms.
- Three surveys and an audit, as part of the understanding of current access to liver services, were conducted using Qualtrics administered by the University of Glasgow.

## Results

### Patient and Public Involvement (PPI) and patient representation

SHARP has made strides to develop projects to improve the care of patients with liver disease. The integral involvement of patients and their representatives is a strong element that has driven this. Specifically, Amy Cordwell from the British Liver Trust was a member of the PMG as a co-applicant, and Kirsty Mills was co-opted into the PAG as a patient member having seen press releases about SHARP. Their input to the project has been invaluable, and their positive impact is best summarised by the quotes they have provided:

*“Being able to participate in the SHARP project has been rewarding for me as a patient. Firstly, I've enjoyed being able to contribute (somewhat!) usefully to discussions, providing my 'lived experience' perspective to flag issues which [healthcare providers] HCPs might not otherwise have considered. It's been exciting to learn about new, exciting technologies which could potentially improve the accessibility of liver disease care for future patients in Scotland. Finally, it's been heartening to witness the positive and proactive attitude with which SHARP members approach this work; it has been clear that members are very passionate about trying to make practical and effective changes to better facilitate patient access across Scotland. This, in turn, gives me a sense of hope for the future.”* Kirsty Mills

*“Participating and being involved with the SHARP project has been an insightful and positive experience. It was heartwarming to see patients being involved from the beginning of this project and gave patients an insight into current research that is being carried out to better the care and treatment of all liver disease patients across Scotland. The patient voice was heard throughout this project and it was brilliant to work alongside such motivated and passionate healthcare professionals to make improvements for liver disease patients for the future”.* Amy Cordwell

In addition to direct patient involvement in the development of projects, we outline below how we sought feedback on the projects that we have developed. We have also promoted the work of SHARP through regular press releases via NHS Research Scotland, which can be accessed via our website: https://www.nhsresearchscotland.org.uk/research-areas/hepatology/sharp. We also promoted our work via our X profile @sharphepatology and presented the project outline as an abstract at the Scotland Health Research and Innovation Conference in October 2023.

### Project development

The purpose of SHARP was to set up and develop projects that identify the barriers faced by both socially and geographically isolated patients when it comes to identifying and treating liver diseases. Early on, we decided that the best approach was to develop separate workstreams to first help identify the issues and then develop projects to tackle them. Below, we outline the four workstreams that we developed and the feedback that we received.

   1. Understanding current access to liver services

Early discussions by the combined PMG and PAG recognised that to address access to hepatology services in Scotland, we would need to identify the barriers to accessing services. There had been a very successful survey conducted by the BLT in 2019 on patients’ attitudes toward disease management. However, there has never been a nationwide survey canvassing not only the opinions of clinicians and patients but also the provision of hepatology services throughout Scotland. Therefore, we devised four separate surveys and audits.

- A patient survey evaluated the interactions with primary and secondary care throughout the patient journey (276 respondents). This was developed in conjunction with BLT.
- Two surveys of the perception of local liver services were completed by primary care physicians (199 respondents) and another by Gastroenterologists/Hepatologists (99 respondents).
- An audit of the hepatology clinical services in each territorial Health Board in Scotland was completed by service providers.

The data from these surveys are currently being analysed and will provide a map of patient and clinician experiences throughout Scotland, and how these experiences correspond to the provision of services nationwide. This will inform the research questions to be addressed, but it will also provide a unique snapshot of the service gaps in Scotland. We plan to publish and present these results as they will be informative for the wider UK hepatology community.

   2. Technologies to monitor and diagnose CLD

Liver disease is often diagnosed through routine blood testing, and many liver diseases require regular monitoring of liver function. Due to its asymptomatic nature, the diagnosis of CLD often relies on opportunistic screening of patients who interact with healthcare through routine blood testing or alternatively portable Fibroscan machines are used in the community outreach setting. CLD is also often diagnosed late because of its relatively asymptomatic disease course. Indeed, up to 61% of patients with steatotic liver disease are diagnosed at the time of decompensation of established cirrhosis ( Hussain *et al.*, 2020). There is no currently available point-of-use (POU) test for liver disease that is simple, rapid, and can be performed in non-clinical settings. Such a test would revolutionise the identification of those at risk of advanced liver disease, as it would enable widespread screening of at-risk populations. Furthermore, many patients live in geographically isolated areas with little access to healthcare services and feedback from BLT patient groups, suggested that the diagnosis or monitoring of liver disease with near-patient testing would dramatically improve patient experience.

To address this unmet need, we have collaborated with Prof. Karen Faulds (University of Strathclyde) and Prof. James Dear (University of Edinburgh), who have already developed a point-of-care assay to detect liver injury in patients who have taken a paracetamol overdose. We propose developing a POU test that quantifies ALT and AST levels using patient finger-prick blood. This assay will enable the immediate identification of liver inflammation as well as the AST:ALT ratio in a non-clinical setting. The Gwent AST project has accurately identified patients at risk of advanced liver disease using an AST:ALT ratio performed by GPs ( Yeoman *et al.,* 2020) and our POU test would take the AST:ALT ratio out of primary care, bringing it into the community, thus enabling cost-effective screening and identification of at-risk populations.

The above is being worked into a grant proposal and if successful, the assay will be developed by Prof. Karen Faulds’ research group and trialled clinically in the hepatology clinics in NHS Tayside.

   3. Identification of patients at risk of liver disease

The median survival of patients with compensated (stable) cirrhosis is ~12 years compared to ~2 years for patients with decompensated cirrhosis ( D’Amico *et al.*, 2006) and only ~21% of patients are diagnosed with advanced liver disease in primary care ( Blais *et al.*, 2015). Therefore, there is a clear benefit of early identification and intervention in patients at the greatest risk of disease progression. The Kerr Report ( NHS Scotland, 2005) stated that the NHS in Scotland should aim to provide continuous, anticipatory care to ensure that, as far as possible health care crises are prevented from happening”. There is already an awareness of the importance of the early detection of asymptomatic advanced liver disease. However, there is also a need to address the detection of early liver disease. This essentially moves the focus of detection ‘upstream’ when interventions may have the greatest impact on subsequent disease incidence. The difficulty, as we have already outlined, is that current community diagnostic strategies rely on patients engaging in healthcare services and undergoing clinical assessments (e.g., blood tests and anthropometric investigations). Therefore, innovative strategies for early recognition and intervention are needed to improve CLD outcomes.

We propose the development of an artificial intelligence tool to predict an individual's risk of being admitted to hospital as an emergency inpatient for chronic liver disease within five years. We aim to develop this tool by using routine electronic healthcare data to predict future liver health. Cognisant that hard-to-reach groups have a paucity of primary care data but do have data capture equity when it comes to secondary care data, we will use data from patients in an unscheduled hospital admission cohort to train and benchmark a collection of classical and state-of-the-art machine learning models on data spanning 10 years to predict the 1-, 3-, and 5-year risks of emergency hospitalisation for liver disease (of any aetiology). We will then aim to validate this tool by working within the Digital & Health Care Wales secure data environment with collaborators from the NIHR-funded Liver Disease Cymru Partnership. One important consideration of such a project is patient and public acceptability, and it will therefore require stakeholder mapping, which will form an integral part of this project, along with the development of an intervention for patients identified as being at high risk. Once validated, this detection methodology will be piloted in different clinical areas to reflect both socially and geographically isolated populations.

We aim to submit this work to the Health and Social Care Delivery Research Call from the NIHR.

   4. Barriers to engagement with care for liver disease

A scoping review conducted by our team in August 2023 identified that did-not-attend (DNA) rates were higher for hepatology than any other specialty and that non-attendance and non-engagement were associated with poorer outcomes, including premature death. The review highlighted a variety of reasons for non-attendance, including the importance of stigma and discrimination as barriers to care, and concluded that there was a paucity of qualitative research specifically exploring barriers to care for people with CLD in the UK. Therefore, we developed a project to explore barriers to care for adults with CLD, and potential ways to overcome these barriers. We achieved this through the following three research objectives:

1. Explore patient and practitioner views and experiences of referral and attendance.
2. Explore views of people at high risk of CLD who are not currently engaged in services.
3. Co-design a suite of recommendations to improve access to and engagement with care for people living with CLD.

People with lived experiences of CLD will be involved in this research, including a PPI co-applicant and peer researchers. Our focus is on the three leading causes of CLD in Scotland: Alcohol-related liver disease, metabolic dysfunction-associated steatotic liver disease (MASLD), and viral hepatitis. We hope that by better understanding the barriers to care, we can improve engagement and outcomes for these patient groups.

We are currently drafting a funding application to submit to the Chief Scientist Office (CSO) for Scotland, specifically for the Health Improvement, Protection, and Services Research Committee.

Workstreams survey

To gauge the relevance of these workstreams, we conducted a survey of patients, caregivers, and allied health professionals. This survey was promoted via social media and administered using Microsoft Forms. 53 people responded to the survey; their backgrounds are outlined in Table 1. 

**Table 1.** Background of survey respondents.

Background	Number of respondents
Person with liver disease	15
A carer for someone with liver disease	1
Primary Care health professional	5
Secondary Care health professional	17
Public Health professional	3
Health and Social Care Partnership professional	4
Other	8

**Figure 1.**
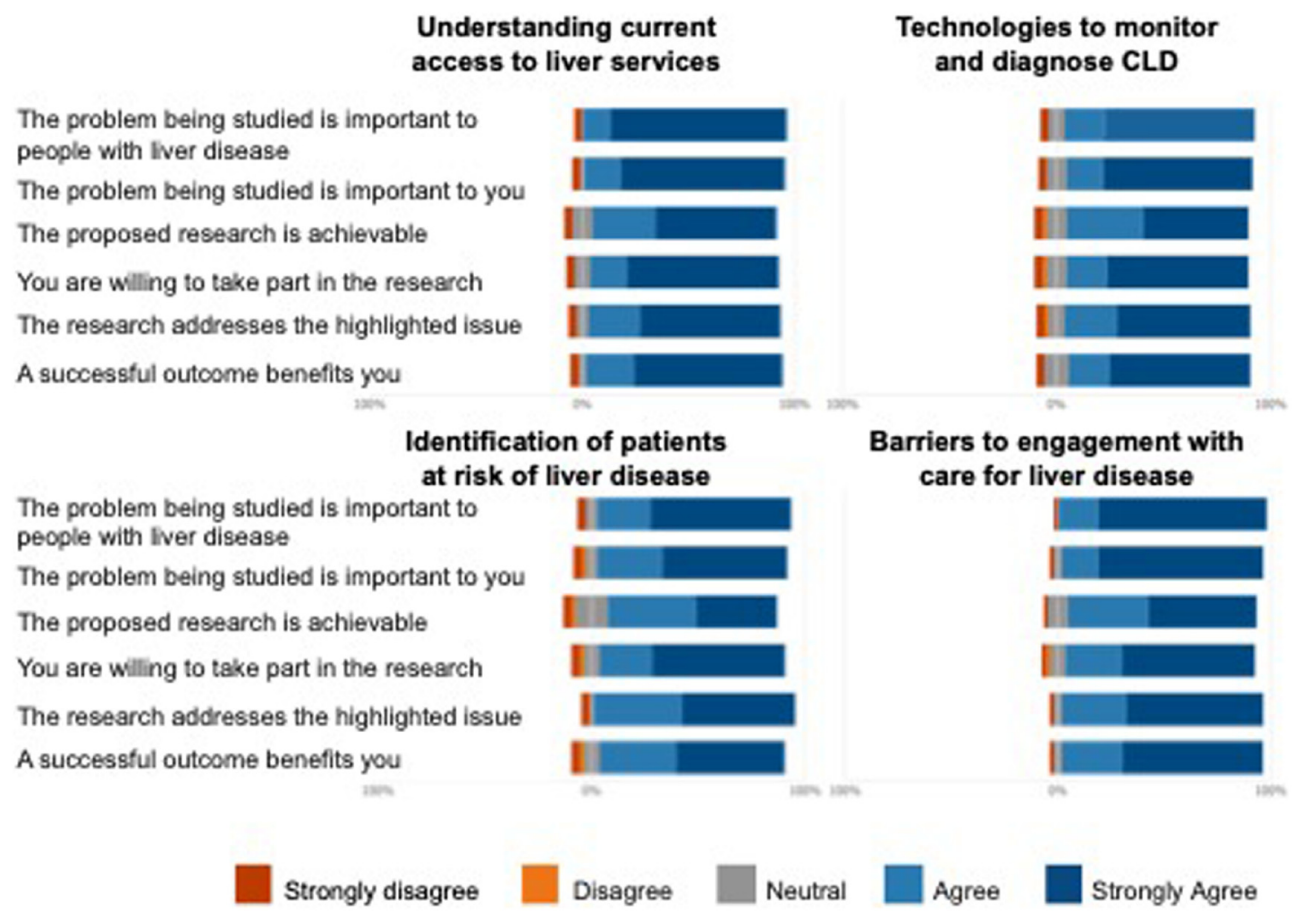
Likert scales of responses to questions relating to the four workstreams developed by SHARP.

Respondents were asked the same set of questions for each workstream and were asked to rate their responses using a Likert scale. The results outlined in Figure 1 demonstrate that the respondents were generally positive about all workstreams. Furthermore, specific comments from respondents helped us refine the project proposals to ensure that all opinions were considered.

## Conclusion

SHARP has made tangible progress towards understanding the issues affecting access to hepatology services in Scotland. The partnership has identified structured workstreams to address these issues and improve the care of patients with liver diseases in Scotland. Through a multidisciplinary pan-Scotland group, we have developed projects that will help identify and break down the barriers that socially and geographically isolated patients face when it comes to identifying and treating liver diseases. We did so by engaging with patients and all relevant stakeholders throughout the partnership. In addition, we performed the first comprehensive national audit and survey of liver services in Scotland encompassing not only an audit of services but also the attitudes of patients and primary and secondary care physicians.

## Ethics and consent

Ethical approval and consent were not required.

## Data Availability

Open Science Framework: DOI: 10.17605/OSF.IO/865WB ( Lynch *et al.,* 2024) This project contains the following underlying data: Data file 1. (Questionnaire_SHARP feedback on proposed research) Data file 2. (Data_SHARP feedback on proposed research) Data are available under the terms of the Creative Commons Zero "No rights reserved" data waiver (CC0 1.0 Public domain dedication).
