# Supplementary material for: The Scottish Hepatology Access Research Partnership (SHARP) improving access to liver services throughout Scotland
Source: NIHR Open Res. 2024 Nov 27;4:58. Originally published 2024 Oct 10. [Version 2] doi: 10.3310/nihropenres.13650.2 (PMC11628933; doi:10.3310/nihropenres.13650.2)
Supplement: Peer review history

# Reviewer report: Reviewer response for version 2

**DOI:** 10.3310/nihropenres.15026.r33852

**Published:** 2024-12-09

**Authors:** Jonathan Soldera

**Affiliations:** 1 Department of Gastroenterology, University of South Wales, Cardiff, Wales, UK

My concerns have been addressed.

Is the work clearly and accurately presented and does it cite the current literature?

Yes

If applicable, is the statistical analysis and its interpretation appropriate?

Yes

Are all the source data underlying the results available to ensure full reproducibility?

Yes

Is the study design appropriate and is the work technically sound?

Yes

Are the conclusions drawn adequately supported by the results?

Yes

Are sufficient details of methods and analysis provided to allow replication by others?

Yes

Reviewer Expertise:

AI, Gastroenterology, Hepatology.

I confirm that I have read this submission and believe that I have an appropriate level of expertise to confirm that it is of an acceptable scientific standard.

# Reviewer report: Reviewer response for version 2

**DOI:** 10.3310/nihropenres.15026.r33853

**Published:** 2024-11-28

**Authors:** Miwa Takao

**Affiliations:** 1 Gifu University, Gifu, Japan

The authors have appropriately revised the manuscript based on the comments. Thank you for the opportunity to review.

Is the work clearly and accurately presented and does it cite the current literature?

Yes

If applicable, is the statistical analysis and its interpretation appropriate?

Yes

Are all the source data underlying the results available to ensure full reproducibility?

Yes

Is the study design appropriate and is the work technically sound?

Yes

Are the conclusions drawn adequately supported by the results?

Yes

Are sufficient details of methods and analysis provided to allow replication by others?

Yes

Reviewer Expertise:

Liver cirrhosis, Alcohol-related liver disease, Metabolic dysfunction-associated steatotic liver disease, Hepatic encephalopathy, Sarcopenia, Frailty

I confirm that I have read this submission and believe that I have an appropriate level of expertise to confirm that it is of an acceptable scientific standard.

# Reviewer report: Reviewer response for version 1

**DOI:** 10.3310/nihropenres.14820.r33239

**Published:** 2024-10-25

**Authors:** Miwa Takao

**Affiliations:** 1 Gifu University, Gifu, Japan

In this manuscript entitled “The Scottish Hepatology Access Research Partnership (SHARP) improving access to liver services throughout Scotland”, the authors developed a research project for better access to the liver unit for patients with chronic liver disease (CLD). While the topic is an important point, there are some concerns which should be clarified.

 Major comments

1. The paper thoroughly describes the socioeconomic challenges faced by CLD patients in Scotland, particularly in rural and deprived areas. The evidence of this manuscript can be more valuable if the authors can provide a deeper discussion of how other countries with similar challenges have addressed such issues.
2. This study is conducted in Scotland where I know that there are many general practitioners (GP) for daily care. Is the GP system provided by the NHS insufficient for patients with CLD? The authors should summarize this point considering the international readers who do not understand the medical system in Scotland.
3. The study highlights the importance of point-of-care tests in CLD. For instance, measurement of ALT and AUDIT questionnaire are useful to identify CLD (Tajirika S, et al., 2023 [Ref 1]). In addition, screening for cognitive impairment, sarcopenia, and frailty are also important in patients with CLD. The authors should further discuss these potential tools in the discussion.
4. The development of an AI tool to predict emergency admissions due to CLD may help patients. However, it difficult to consider that patients who do not have good access to the liver unit, such as homeless, has good access to these tools as well. The authors should discuss how to apply these models to such potential patients with CLD.

Is the work clearly and accurately presented and does it cite the current literature?

Yes

If applicable, is the statistical analysis and its interpretation appropriate?

Yes

Are all the source data underlying the results available to ensure full reproducibility?

Yes

Is the study design appropriate and is the work technically sound?

Yes

Are the conclusions drawn adequately supported by the results?

Yes

Are sufficient details of methods and analysis provided to allow replication by others?

Yes

Reviewer Expertise:

Liver cirrhosis, Alcohol-related liver disease, Metabolic dysfunction-associated steatotic liver disease, Hepatic encephalopathy, Sarcopenia, Frailty

I confirm that I have read this submission and believe that I have an appropriate level of expertise to confirm that it is of an acceptable scientific standard, however I have significant reservations, as outlined above.

## References

[1] : Usefulness of health checkup for screening metabolic dysfunction-associated fatty liver disease and alcohol-related liver disease in Japanese male young adults. *Sci Rep* .2023;13(1) : 10.1038/s41598-023-34942-x 7987 10.1038/s41598-023-34942-x 37202437 PMC10195773

# Response

**Published:** 2024-11-25

**Authors:** Ruairi Lynch

**Affiliations:** NHS Tayside, Dundee, Scotland, UK

We thank the reviewer for their comments. We have responded to their numbered comments below:

1. The paper focuses on Scotland as it is the report for an NIHR project that was funded to set up a network looking at barriers to provision of care in Scotland. We have referenced the reviewer’s paper highlighting that some countries use routine medical check-ups to identify not only liver disease but also other medical conditions.
2. Rather than insufficiencies in GP services, there is greater understanding that it is the recognition of liver disease which is deficient across all services. Therefore, we sought to address this with technologies to monitor and diagnose CLD and identification of patients at risk of liver disease workstreams. Provision of GP services may be more challenging depending upon geographical circumstances, and we have now further emphasised this in the introduction.
3. We note the reviewer’s interesting paper, there may be a role for performing AUDIT questionnaires alongside POU tests. Again, whilst this might address a common cause of liver disease in Scotland, it is not the purpose of the POU assay to be disease specific. Furthermore, we are in the development phase of the POU assay and any trial in patients will be for validation. It may be that once validated there may be applications that would use the POU test in conjunction with other assessments. Screening for cognitive impairment, sarcopenia, and frailty are indeed important to patients with established liver disease, however are less relevant to the SHARP workstreams which are targeting identification of early CLD.
4. The AI tool will be initially validated in GP practices but it will be developed with and use routinely available secondary care data to identify patients that don’t engage routinely with medical services apart from during crises. This means that those who do not engage with their GP can still be identified. There is always a challenge in getting these patients to engage, and this will be particularly evident if we identify those at risk. The acceptability of this approach forms a mainstay of our assessment and is why we are also looking into the barriers to engagement. Furthermore, in Scotland there are practices that cater to the homeless population where access is facilitated by not requiring a registration, it may be that we target these practices once the tool is validated.  We have amended the text to reflect these challenges.

# Reviewer report: Reviewer response for version 1

**DOI:** 10.3310/nihropenres.14820.r33116

**Published:** 2024-10-14

**Authors:** Jonathan Soldera

**Affiliations:** 1 Department of Gastroenterology, University of South Wales, Cardiff, Wales, UK

This study investigates the Scottish Hepatology Access Research Partnership (SHARP), which aims to improve access to liver services in Scotland, particularly in addressing the socioeconomic and geographic challenges that hinder care for chronic liver disease (CLD) patients. The authors utilize surveys, audits, and the development of new technologies, including AI tools, to enhance diagnosis and predict emergent admissions in CLD patients. SHARP focuses on improving patient engagement and care delivery for individuals living in isolated regions.

 This paper presents a very interesting and important project. The initiative to use AI to predict CLD outcomes and improve the quality of liver transplantation (LT) services is commendable and has the potential to make a significant impact: Soldera J, et al., 2024 (Ref 1) ; Chongo G, et al., 2024 (Ref 2)

 However, I believe the authors should also address the need for psychiatric support and addiction therapy, such as Alcoholics Anonymous, in the treatment of alcoholism, which remains the major cause of chronic liver disease in Scotland.

 Incorporating mental health and addiction services into the SHARP framework would provide a more comprehensive approach to treating CLD and improving long-term outcomes. Additionally, it would be beneficial to see how SHARP plans to integrate these services alongside their technological and clinical interventions.

Is the work clearly and accurately presented and does it cite the current literature?

Yes

If applicable, is the statistical analysis and its interpretation appropriate?

Yes

Are all the source data underlying the results available to ensure full reproducibility?

Yes

Is the study design appropriate and is the work technically sound?

Yes

Are the conclusions drawn adequately supported by the results?

Yes

Are sufficient details of methods and analysis provided to allow replication by others?

Yes

Reviewer Expertise:

AI, Gastroenterology, Hepatology.

I confirm that I have read this submission and believe that I have an appropriate level of expertise to confirm that it is of an acceptable scientific standard, however I have significant reservations, as outlined above.

## References

[1] : Predicting major adverse cardiovascular events after orthotopic liver transplantation using a supervised machine learning model: A cohort study. *World J Hepatol* .2024;16(2) : 10.4254/wjh.v16.i2.193 193-210 10.4254/wjh.v16.i2.193 38495288 PMC10941741

[2] : Use of machine learning models for the prognostication of liver transplantation: A systematic review. *World J Transplant* .2024;14(1) : 10.5500/wjt.v14.i1.88891 88891 10.5500/wjt.v14.i1.88891 38576762 PMC10989468

# Response

**Published:** 2024-11-25

**Authors:** Ruairi Lynch

**Affiliations:** NHS Tayside, Dundee, Scotland, UK

We thank the reviewer for their comments and we have outlined responses below paying credence to the fact that the project has now concluded. With regards to the comments on alcoholism; although alcohol excess remains a significant issue within Scotland the focus of SHARP was reaching out to populations that are socially and geographically isolated. Other NIHR partnerships have been developed that have a disease specific approach, which we tried to avoid. We are certain that our AI project will identify a variety of aetiological factors that require interventions. The suite of interventions that we will use will be tailored to patients and cover Alcohol amongst others. The project looking at barriers to engagement will also undoubtedly identify that there are multiple barriers to engagement and alcohol may play a role here. We have now emphasised in the text that interventions will include those focusing on a variety of aetiologies including alcohol. With regards to incorporating mental health and addiction services; SHARP included representatives from alcohol and drug partnerships throughout Scotland both in the PMG and the PAG. These members have been integral in developing projects and indeed are involved in the subsequent applications that have been developed from the initial project. We have edited the text to emphasise their involvement in the PMG and PAG. With regards to the integration of these services into our technological and clinical interventions. The suite of projects proposed by SHARP endeavour to identify both patients at risk of liver disease and the barriers to engagement with liver services. We have outlined above that we will have integrated interventions for patients but the exact interventions and who will be involved depend on the populations and challenges identified. Integration of mental health and addiction services into downstream interventions will likely form part of future projects.
